# Supplementary material for: Genetic Screen in Adult Drosophila Reveals That dCBP Depletion in Glial Cells Mitigates Huntington Disease Pathology through a Foxo-Dependent Pathway
Source: Int J Mol Sci. 2021 Apr 9;22(8):3884. doi: 10.3390/ijms22083884 (PMC8069648; doi:10.3390/ijms22083884)
Supplement: Supplementary file 1 [file ijms-22-03884-s001.zip › ijms-1110775-sup-original/supplementary figures.pdf]

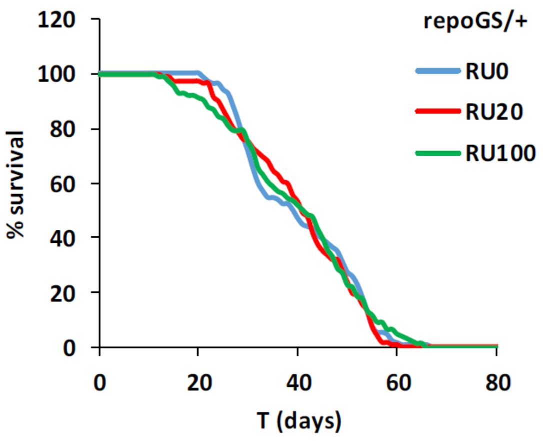

Fig.S1: Induction of repoGS is not toxic in Drosophila glial cells. Progeny issued from a cross between the GeneSwitch repoGS line and a *w1118* control line was dispatched at emergence on fly food containing different RU486 concentrations (RU0: 0µg/ml; RU20: 20µg/ml; RU100: 100µg/ml) and their lifespan was assessed. No statistically significant variations were detected between survival curves (pLogRank>0.5; n>120).

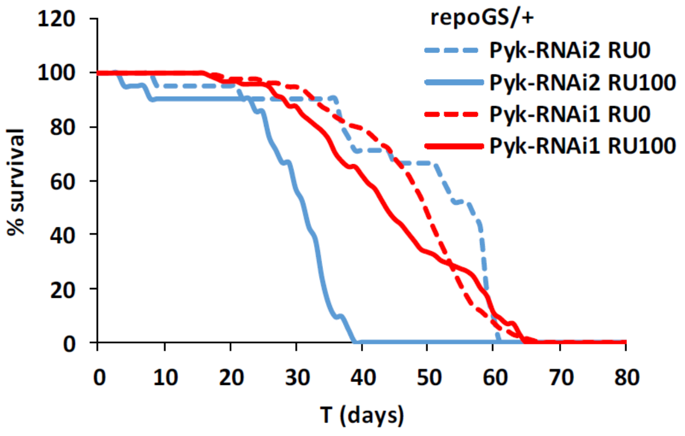

Fig.S2: Depletion of Pyk in glial cells may be deleterious. A repoGS line was crossed with two independent RNAi lines modulating Pyk expression (see TabS4 for detailed genotypes). Lifespan of progeny expressing (RU100) or not (RU0) the RNAis were compared to evaluate potential toxicities. Under these conditions, no toxicity was observed with the RNAi1 transgene (MLS= 47.8, 43.9; n= 136, 98 for RU0 and RU100 respectively; pLogRank>0.05) while a strong deleterious effect is observed with the RNAi2 transgene (MLS= 48.4, 28.3; n= 21, 21 for RU0 and RU100 respectively; pLogRank<10^{-7}).

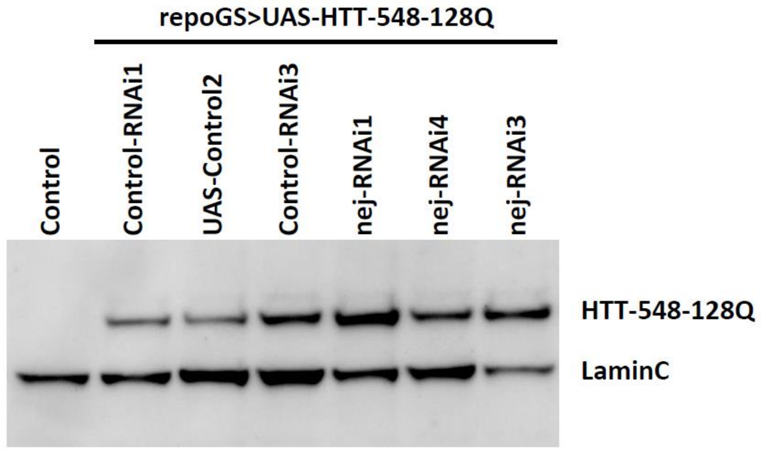

Fig.S3: No evidence of reduced expression of mHTT after dCBP depletion. The level of expression of Huntingtin in repoGS>UAS-HTT-548-128Q flies co-expressing RNAi constructs targetetting dCBP or control transgenes, was analyzed by Western blot, with antibody directed against LaminC as a loading control. The specificity of the human HTT antibody was checked in a *w1118* strain. Overall, the data does not indicate a reduced expression of mHTT after dCBP depletion. See TabS4 for detailed genotypes.

Tab. S1 Genes analyzed. The fly symbols of the genes analyzed in this study is provided in column 2 and are preceded, in column 1, by the type of gene, and followed in column 3 by the FlyBase gene identifier. The best human orthologue of the fly gene, as determined by the DIOPT software (https://www.flyrnai.org/cgi-bin/DRSC_orthologs.pl) is provided in column 4 and is followed, in column 5, by the DIOPT weighted score for the quality of the homology.

Tab. S2 Full results of the screen. The fly symbols of the genes analyzed in this study is provided in column 2 and are preceded, in column 1, by the type of gene. The genotype analyzed is given in column 3, followed by the number of independent experiment N, the mean of the mean lifespan (MLS, column 5), the mean of the ratio of MLS to control MLS (column 6) and the standard deviation of this ratio when N>1 (SD, column 7).

Tab. S3 Modifiers identified in the screen. The fly symbols of the genes analyzed in this study is provided in column 2 and are preceded, in column 1, by the type of gene. The genotype analyzed is given in column 3. In column 4 is indicated whether the transgene leads to loss of function (LOF) or gain of function (GOF) of the gene. The mean ratio of MLS between flies of the indicated genotype and flies of a control genotype is given in column 5.

Tab. S4 Correspondence between figure legends and full genotypes. Identification of the figures is indicated in the first column. Legends that are present in the figure are given in column 2, followed by the full genotypes to which they refer. In column 3 is indicated whether the transgene is induced (RU100) or not (RU0) in the experimental condition.
